# Morphology-Controlled *Green* Synthesis of Magnetic Nanoparticles Using Extracts of ‘Hairy’ Roots: Environmental Application and Toxicity Evaluation

**DOI:** 10.3390/nano12234231

**Published:** 2022-11-28

**Authors:** Natalia Kobylinska, Dmytro Klymchuk, Olena Khaynakova, Volodymyr Duplij, Nadiia Matvieieva

**Affiliations:** 1 Dumansky Institute of Colloid and Water Chemistry, National Academy of Science of Ukraine, 42 Akad. Vernadskoho Blvd., 03142 Kyiv, Ukraine; 2 Kholodny Institute of Botany, National Academy of Science of Ukraine, 2 Tereshchenkivska Str., 02000 Kyiv, Ukraine; 3 Faculty of Chemistry, University of Oviedo, 8 Julián Claveria Av., 33006 Oviedo, Spain; 4 Institute of Cell Biology and Genetic Engineering, National Academy of Science of Ukraine, 148 Zabolotnogo Str., 03143 Kyiv, Ukraine

**Keywords:** *Artemisia tilesii* L. “hairy” roots, magnetite nanoparticles, biosynthesis, magnetic separation, toxic effect

## Abstract

Magnetic nanoparticles (MNPs) were “*green*” synthesized from a FeCl_3_/FeSO_4_/CoCl_2_ mixture using ethanolic extracts of *Artemisia tilesii* Ledeb ‘hairy’ roots. The effect of chemical composition and reducing power of ethanolic extracts on the morphology, size destribution and other features of obtained MNPs was evaluated. Depending on the extract properties, nanosized magnetic materials of spherical (8–11 nm), nanorod-like (15–24 nm) and cubic (14–24 nm) shapes were obtained via self-assembly. Microspherical MNPs composed of nanoclusters were observed when using extract of the control root line in the synthesis. Polyhedral magnetic nanoparticles with an average size of ~30 nm were formed using ‘hairy’ root ethanolic extract without any additive. Studied samples manifested excellent magnetic characteristics. Field-dependent magnetic measurements of most MNPs demonstrated a saturation magnetization of 42.0–72.9 emu/g with negligible coercivity (∼0.02–0.29 emu/g), indicating superparamagnetic behaviour only for solids with a magnetite phase. The synthesized MNPs were minimally aggregated and well-dispersed in aqueous medium, probably due to their stabilization by bioactive compounds in the initial extract. The nanoparticles were tested for magnetic solid-phase extraction of copper (Cu), cadmium (Cd) and arsenic (As) pollutants in aqueous solution, followed by ICP-OES analysis. The magnetic oxides, mainly magnetite, showed high adsorption capacity and effectively removed arsenic ions at pH 6.7. The maximum adsorption capacity was ~150 mg/g for As(III, V) on the selected MNPs with cubic morphology, which is higher than that of previously reported adsorbents. The best adsorption was achieved using Fe_3_O_4_-based nanomaterials with low crystallinity, non-spherical form and a large number of surface-localized organic molecules. The phytotoxicity of the obtained MNPs was estimated in vitro using lettuce and chicory as model plants. The obtained MNPs did not exhibit inhibitory activity. This work provides novel insights on the morphology of “*green*” synthesized magnetic nanoparticles that can be used for applications in adsorption technologies.

## 1. Introduction

Water pollution by toxic inorganic or organic compounds is a considerable global problem because of the adverse effects of such compounds on the environment and human health, even at very low concentrations [1,2]. It is estimated that more than 150 million people worldwide are exposed to potentially toxic elements such as cadmium (Cd), copper (Cu) and arsenic (As) at levels higher than those recommended by WHO (for example, 10 μg/L for Cd and As in drinking water) [2,3,4] because these pollutants are present in both underground and surface water sources [5]. Industrial and agricultural discharge and urban activity wastewaters are important anthropogenic sources that contribute to the increasing amount of toxic compounds in the environment. As some pollutants are not biodegradable, they can be accumulated in living organisms and are toxic to aquatic flora and fauna, as well as to humans [6]. Regarding toxicity, e.g., As(III) is 60-fold more toxic than As(V). Furthermore, contamination of water medium by phosphate (P(V)) is known to be the main obstacle to remediation methods. Therefore, in order to prevent excessive concentrations of As(III, V), P(V) and heavy metal ions in drinking water, innovative and economically justified treatment methods are urgently recommended for the effective removal of inorganic toxicants from polluted water sources. 

The necessity for modern science and technology to identify novel materials with specific properties has increased the interest of the global industrial and scientific community in nanomaterials. Advances in nanoscience and nanotechnology have brought about the need to develop environmentally friendly nanoparticle preparation processes. Owing to their properties, magnetic nanoparticles (MNPs) have received considerable attention for use in various applications, especially in the biomedical and ecoanalytical fields [7]. In recent decades, a large amount of scientific data on the preparation of MNPs has been published, enabling the development of synthesis processes for MNPs with the required morphological, physical and chemical properties. Among magnetic materials, iron oxides [8] such as magnetite (Fe_3_O_4_), hematite (α-Fe_2_O_3_), maghemite (γ-Fe_2_O_3_), bernalite (Fe(OH)_3_), β-Fe_2_O_3_, akaganeite (β-FeOOH), FeO and green rust (Fe(II)/Fe(III)-LDH) have attracted considerable attention, owing to their unique properties, including biocompatibility and high magnetic permeability, combined with cost-effective synthesis, chemical stability and low toxicity toward human cells [9]. Inorganic materials such as radioactive (^234,235,238^U(VI), ^137^Cs(I), etc.) and heavy metal ions (Cr(III), Cu(II), Pb(II), etc.) can be effectively removed using magnetic nanoparticles. In addition, nutrients can be recovered and pollutants can be detected in waste effluents using magnetic nanoparticles. Generally, Fe(III)-based hydroxides and corresponding oxides (ferrihydrite, goethite, feroxyhyte and hematite) attract special attention owing to their heavy metals adsorption performance in oxy-type and the convenience of magnetic separation [10]. The binding of arsenic with Fe(III)-(hydr)oxides is strongly determined by the type of formed surface complexes and the presence of competitive ions in the solution (for example, phosphate) [11,12]. However, the internal mechanism of the formation of such nanoparticles has not been intensively investigated, and only a few papers have been published that clearly focused on the mutual interference and competition of various oxyacid-type heavy metal pollutants in a coexisting system during adsorption processes using MNPs as adsorbents.

Owing to their small size (~10 nm), magnetic nanocrystals have the advantage of superparamagnetism, but the weak magnetic response limits their practical applications due to low absorption capacity and specific surface area. Miscellaneous methods such as the microemulsion technique, the electrochemical route, sonochemical synthesis, coprecipitation, and hydrothermal- and microwave-based procedures have been developed to produce MNPs. Various reviews devoted to the techniques mentioned above have been published [13,14]. However, these methods are subject to several limitations, such as difficulties with respect to operating conditions, generation of hazardous waste in the synthetic process and the frequent use of highly biotoxic products that could cause significant environmental risks. Magnetite nanoparticles can be easily oxidized to ferrimagnet maghemite or other iron oxide phases [15]. An experimental challenge in the synthesis of Fe_3_O_4_ by coprecipitation is to control the particle size and thus achieve a narrow particle size distribution. Nanoparticles prepared by simple traditional coprecipitation method tend to be rather polydisperse. It is well-known that a short burst of nucleation and subsequent slow, controlled growth is crucial to produce monodisperse particles. Controlling these processes is therefore the key factor in the synthesis of monodisperse iron oxide magnetic nanoparticles. For larger sizes, the magnetic moment remains fixed, resulting in stable single-domain ferrimagnetic behavior. However, for polydisperse (large and small) magnetite crystals, multiple magnetic domains of opposite polarity are formed in individual crystals, which reduces their magnetostatic energy. This phenomenon is also observed in other spinel ferrites, resulting in a significantly reduced magnetization saturation of MNPs in the multidomain regime.

So-called “green” synthesis enables the attainment of MNPs characterized by low toxicity and safety for the human organism because of application of nontoxic plant extracts for nanoparticle initiation [16]. In this context, methods for the synthesis of green MNPs represent an interesting option owing to the formation of an external organic layer of MNPs, which acts as a capping agent. Biosynthesized MNPs are a practical alternative to a chemical rout based on the use of plant extract, with several specific features in their chemical and physical properties [13,17,18]. Plant extracts containing flavonoids, polyphenols, sugars and aromatic compounds can reduce iron, leading to the formation of Fe_3_O_4_ nanoparticles due to some components present in the extract or to the interaction between compounds in the bioreaction mixture, e.g., ascorbic acid [19], amino acids [20], starch [21], glucose and gluconic acid [22]. Thereby, these components are able to improve the properties of the nanoparticles. The main advantages of biosynthesized MNPs are related to low-cost functionalization, environmental friendliness and high biological compatibility [16]. It has been observed that “green” synthesis is able to improve the dispersity and chemical stability MNPs, enhance biocompatibility and reduce toxic effects compared to other nanoparticles coated with synthetic organic layers [23]. Remarkably, the formation of an organic layer in this type synthesis leads to avoidance of undesired agglomeration and maintains the colloidal stability of the nanoparticles to increase their efficiency in diluted systems. Therefore, the focus of this study was the features of MNPs with various morphologies prepared through “green” synthesis. 

Multiple plants, including Hordeum vulgare [24], Yarrowia lipolytica [25], Averrhoa carambola [26], green tea [27], Euphorbia cochinchinensis [28], etc., have been used for preparation of extracts for biosynthesis of MNPs. The production of spherical and fairly uniform nanoparticles is an attractive feature of some of the synthetic methodologies published recently. Dharmarajan et al. [28] reported the preparation of spherical MNPs using E. cochinchinenensis extracts. These extracts contained phenols and flavonoids capable of acting as reducing agents for the initiation of Fe_3_O_4_ nanoparticle formation. The obtained Fe_3_O_4_ NPs were used for the removal of doxorubicin from real wastewater with an efficiency of 80%. Nikić and coauthors [29] proposed the preparation of Fe_3_O_4_ nanoparticles through a one-pot reaction using onion peel and corn silk extracts. Although no magnetization studies were reported, evidence of the magnetic response of the prepared NPs was discussed [29]. A. carambola leaf extract was used to prepare monodisperse magnetite nanoparticles (46 ± 2 nm) and a composite with graphene oxide (g-Fe_3_O_4_/2RGO), with a wide spectrum of applications, such as photocatalytic performance for reducing Cr(VI) (until 97%), degradation of phenolic compounds and antibacterial activity against Gram-positive and Gram-negative bacteria [26]. Another ‘green’ approach was used for the synthesis of magnetite with Cynara cardunculus leaf extract [30]. Hemispherical and agglomerated nanoparticles were synthesized. For the synthesis, different parts of the plants (leaves, flowers, seeds and fruits) were used for extract preparation. The use of ecological/biocompatible plant extracts to coat MNPs such nanoparticles suitable for a wide variety of environmental applications. Most of MNPs biosynthesized by different extracts are spherical or hemispherical in shape. Only a few reported procedures have led to the synthesis of non-spherical nanoparticles. For example, Stan and co-authors reported the preparation of MNPs using aqueous peel extracts of Citrus limon, Vitis vinifera and Cucumis sativus agro-waste [31]. Micrographs of the MNPs revealed the presence of aggregates with varied shapes and sizes, i.e., a mixture of spherical particles with other morphologies. In [32], Sebastian et al. proposed the preparation of magnetite nanoparticles using a coconut husk extract from Cocos nucifera. Coconut husk extract is rich in benzoic and caffeic acids, which may act as reducing agents of Fe(III) and as a stabilizer during the formation of MNPs with irregular morphology and non-uniform sizes. A maximum adsorption capacity to remove Cd(II) from aqueous solutions of 9.6 mg/g was achieved. Psidium guavaja-Moringa oleifera aqueous leaf extract was used to prepare MNPs [33]. This methodology led to the formation of samples with low-saturation magnetization (5.87 emu/g). MNPs showed considerable potential to inhibit bacterial growth and degrade dyes, owing to the variety of functional groups on the surface of the colloidal nanoparticles. Thus, biosynthesis of MNPs could represent an alternative method for nanoparticles synthesis without the use of special equipment and toxic chemicals [34]. Therefore, there is lack of information about the key components of plant extracts for the successful synthesis of MNPs with high yield and regulated morphology.

Plant genetic transformation by Agrobacterium rhizogenes is a method used to obtain “hairy” root cultures [35]. It is also possible to obtain samples of “hairy” roots characterized by the increased reducing power owing to the incorporation of bacterial rol genes into the plant genome. The presence of these genes in plant cells can lead to the activation of the synthesis of secondary metabolites with high bioactivity. Identification of the key components in this process is significant study and can provide additional information to support the determination of optimal conditions for effective formation of MNPs with enhanced features.

Recently, the use of *Artemisia annua* L. “hairy” root extract was reported as an easy synthetic method for the preparation Fe_3_O_4_ and CoFe_2_O_4_ [36]. The ‘hairy’ root extract rich in polyphenolic compounds allowed for the creation of the technology for the formation and stabilization of MNPs. The obtained MNPs showed a dual functionality and were able to remove 88% of the Cu(II) ions. The compositions of the phases, the size of the particles and the adsorption properties of these materials are likely dependent on the components of the extracts and their relationships, which is why the biosynthesis conditions should be further optimized and investigated.

Accordingly, the aim of the present work was to develop and optimize the synthesis of MNPs using *Artemisia tilesii* “hairy” roots, as well as to study the effect of the type of plant extract on the morphology of MNPs formed at a room temperature. Possibilities of “green” synthesis of MNPs using ethanol extracts of A. tilesii “hairy” roots are reported herein. The effect of the chemical composition and reducing activity of the extracts on the process of MNPs synthesis with varying morphology and size is also discussed. The as-prepared MNPs were characterized using miscellaneous advanced instrumental techniques. MNPs were employed for the removal of inorganic cationic and anionic species (Cu(II), Cd(II), As(III, V) and P(V)) in aqueous solution. Furthermore, the adsorption mechanisms are discussed based on batch experimental data. The remediation procedure must be truly sustainable and environmentally friendly. To this end, the phytotoxicity of MNPs was examined for safe application.

## 2. Materials and Methods

### 2.1. Extract Preparation

**Figure 1:**
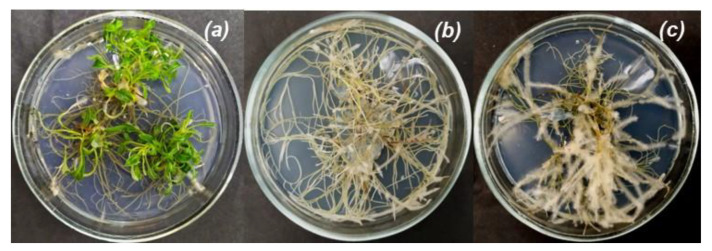
*Artemisia tilesii* control plants grown in vitro (**a**) and various lines of “hairy” root culture (**b**—№ 4, **c**—№ 3).

*Artemisia tilesii* L. “hairy” roots from the collection of the Institute of Cell Biology and Genetic Engineering NAS of Ukraine were grown using Murashige and Skoog solidified nutrient medium (Duchefa, Haarlem, The Netherlands) with twice-reduced concentration (½MS) in vitro. The control mother plants were cultivated under the same conditions (Figure 1). 

Transgenic and control lyophilized samples were used for extract preparation (Figure 1). Briefly, 50 mg of dried roots and leaves of the control plants (№ 1 and 2) and “hairy” roots of A. tilesii (clone №s 3, 4, 5, 6, 7 and 8) were powdered by Retsch MM400 (Germany). The obtained powder was added to 5 mL of extraction solvent (70 vol.% EtOH (Sigma-Aldrich, HPLC grade)) and stirred on a rotary shaker (Clim-O-Shake system Kuhner IRC-1-U) at 28 °C for three days. Then, the extracts were centrifuged (Eppendorf^®^ Microcentrifuge 5415C, München, Germany) at 14,000 ppm for 5 min. The supernatants were used for the biosynthesis of MNPs.

### 2.2. Synthesis of MNPs

Initially, 2.0 mL of 5.4% FeCl_3_ (FeCl_3_·6H_2_O, 97%, Merck), 2.0 mL of 2% FeSO_4_ (FeSO_4_·7H_2_O (Alfa Aesar, Haverhill, MA, USA, ≥98%) and 0.2 mL of 1% CoCl_2_ (CoCl_2_·2H_2_O (Alfa Aesar, ≥98%) were mixed with 1 mL of as-prepared extracts. The obtained mixture was kept at room temperature for 30 min without stirring. Subsequently, the pH was adjusted to 9 using NH_3_∙H_2_O (Alfa Aesar, 10%). The resulting suspension turned black and acquired magnetic properties. The as-prepared MNPs solids were magnetically separated from the liquid phase to acquire black solids by applying a NdFeB magnet. Then, magnetic solid MNPs were sequentially washed with deionized water once and ethanol thrice. The final solids were vacuum-dried for 24 h at 30 °C.

Black solid samples obtained using roots and leaves from the control plants (№ 1 and 2) and “hairy” roots (clone №s 3, 4, 5, 6, 7 and 8) of *A. tilesii* were labelled in as MNP-1, MNP-2, MNP-3, MNP-4, MNP-5, MNP-6, MNP-7 and MNP-8. All prepared MNPs were characterized using multiple advanced instrumental techniques.

### 2.3. Characterization of MNPs

Powder X-ray diffraction (PXRD) was performed on a PANalytical X’Pert Pro diffractometer with a X’Celerator detector using Cu Kα radiation (50 kV, 40 mA). The average size of the crystalline was estimated using Scherrer’s equation [37]. 

X-ray photoelectron spectroscopy (XPS) measurements were performed with an ESCALAB 220 XL spectrometer from Vacuum Generators featuring a monochromatic Al Kα X-ray source (1486.6 eV) and a spherical energy analyzer operated in CAE (constant analyzer energy) mode (CAE = 100 eV for survey spectra and CAE = 40 eV for high-resolution spectra) using the electromagnetic lens mode. The surface was prepared by depositing 100 µL of the particle suspension (1 mg/mL) in water onto silicon wafers and allowed to dry. The detection angle of the photoelectrons was 30°, as referenced to the sample surface. The XPS spectra were corrected according to the binding energies of Au 4f 7/2, equal to 80.0 eV.

Transmission electron microscopy (TEM) was used to examine the size and morphology of the biosynthesized MNPs. TEM images of the MNPs were recorded using a JEM-I230 (JEOL, Tokyo, Japan) operating at an accelerating voltage of 80 kV. TEM grids were preliminarily supplied by a formvar film, which was then fixed by carbon using a JEE-4X vacuum evaporator (JEOL, Tokyo, Japan). Small (0.01–0.05 μL) drops of the MNP solutions were applied to copper grids under a light microscope (MBS-9, USSR) and dried in air at room temperature.

Fourier transform infrared (FTIR) spectra were recorded using a Nicolet 470 Nexus instrument (Thermo Scientific, Waltham, MA, USA). The data were processed so that each final spectrum represents an average of 32 separate scans in KBr pellets. Dried MNPs (1 mg) were mixed with KBr powder (100 mg) in an agate mortar. The signal formed in air was subtracted as the background.

Field-dependent magnetic measurements were performed with an EV9 vibrating-sample magnetometer under in high-sensitivity reciprocal space mode. In order to determine the saturation magnetization (Ms), magnetic hysteresis loop experiments were performed in a magnetic field (H) of 20 kOe. 

X-ray photoelectron spectroscopy (XPS) measurements were performed with an ESCALAB 220 XL spectrometer from Vacuum Generators featuring a monochromatic Al Kα X-ray source (1486.6 eV) and a spherical energy analyzer operated in constant analyzer energy mode (100 eV for survey spectra and 40 eV for high-resolution spectra). The surface was prepared by depositing 100 µL of the MNP suspension (1 mg/mL) in ethanol followed by drying.

In order to precisely evaluate the Fe/Co ratio in the obtained materials, inductively coupled plasma optical emission spectrometry (ICP-OES, iCAP 6500 Duo ICP, Thermo Fisher Scientific) was used after prior digestion of solids in concentrated HNO_3_. Elements in solution were quantified after total dissolution of MNPs and measured at the following emission wavelengths: 259.837 nm (Fe II) and 238.892 nm (Co).

### 2.4. Flavonoid Content Assay

Total flavonoid content was determined using a modified method [38]. Briefly, the extract (0.25 mL) was mixed with double-distilled water (1 mL) and 0.075 mL of NaNO_2_ (5%) solution. Then, 0.075 mL of AlCl_3_ (10%) was added to the reaction mixture. In the next step, 0.5 mL of NaOH (1M) solution and double-distilled water (0.6 mL) were added. The obtained solution was mechanically mixed. The absorbance of the obtained mixture was measured at 510 nm using a UV-Vis spectrophotometer (Fluorat-02 Panorama, St.Petersburg, Russia). The flavonoid content was calculated according to the calibration plot (A_510_ = 1.305, Crutin, R^2^ = 0.9687) and expressed in rutin equivalents (mg (RE)/g).

### 2.5. Reducing Power Assay

A traditional method [39] was used to determine the ability of extracts to reduce ferric ions. Briefly, the ethanol extracts (0.016–0.125 mL) were added to 0.3 mL of phosphate buffer (pH 6.6) and 0.3 mL of potassium ferricyanide (1%), mixed thoroughly and incubated at 50 °C for 30 min. After this procedure, 0.3 mL of trichloroacetic acid (10%) was added. Then, 1.25 mL of the solutions was mixed with 1.25 mL of double-distilled water and 0.25 mL of FeCl_3_ (0.1%). Absorbance was determined spectrophotometrically at 700 nm (Fluorat-02 Panorama, Russia). Reducing power expressed as equivalent concentrations (EC0.5) was determined as the amount of dry root material necessary to achieve absorbance (A_700_) = 0.5. Rutin solution (1 mg/mL) was used as a positive control. 

### 2.6. Phytotoxicity Assessment of MNPs

The inhibitory potential of the obtained MNPs was assessed by phytotoxicity studies with two plant species, i.e., *Cichorium intybus* L. (“*Palla Rossa*”) and *Lactuca sativa* L. (“*Izumrudny*”). Germination tests were conducted by incubation of 100 test seeds in 2 mL of prepared aqueous suspension of MNPs for 2 h. Then, the seeds were transferred to Petri dishes with filter paper and 8 mL of water. Control seeds were placed in Petri dishes without previous MNP treatment. The dishes were then placed in a thermostat at 24 °C. After seed germination, the sprouted seeds were counted, and the weights of seedlings were measured. Nanoparticles were considered non-toxic if the weight of the MNP-treated plants was comparable with the weight of the control plants.

### 2.7. Adsorption Study

Cu(II) and Cd(II) stock solutions were prepared by directly dissolving the appropriate amount of Cu(NO_3_)_2_∙5H_2_O (≥99.0%, Sigma-Aldrich, St. Louis, MI, USA) and Cd(NO_3_)_2_∙4H_2_O (≥98.0%, Sigma-Aldrich, St. Louis, MI, USA) salts, respectively. Stock solutions of arsenic species As(V) (arsenate, AsO_4_^3−^) and As(III) (arsenite, AsO_3_^3−^) were prepared at a concentration of 150 mg/L by dissolving appropriate quantities of sodium arsenate dibasic salt heptahydrate (Na_2_HAsO_4_·7H_2_O, purity ≥ 98.0%, Sigma-Aldrich, St. Louis, MI, USA) and sodium (meta)arsenite (NaAsO_2_, purity ≥ 90.0%, Sigma-Aldrich, St. Louis, MI, USA). The working solutions were prepared by diluting the stock solutions. All solutions were prepared using deionized water (18.2 μS/cm) from a Milli-Q Element System (Millipore, Molsheim, France). The pH of the solutions was measured using a Mettler Toledo pH meter (Seven Compact S210).

Batch adsorption experiments were used to determine the removal efficiency of MNPs toward Cu(II), Cd(II) and As(III, V) ions. Briefly, a fixed quantity (0.1 g) of MNPs was added to 10 mL of 5 mg/L of As(V) at fixed pH in a centrifuge tube and left for 1.0 h to achieve the equilibrium state. Several primary concentrations of As(III, V) in the range of 0.5–150 mg/L were used in onset experiments. The suspension was continuously stirred (230 rpm) using an orbital shaker (Biosan PSU-20i) at room temperature (20 °C). The 2 h reaction time was assumed to be adequate to attain equilibrium for further applications in water treatment technologies. Sorption experiments were conducted in the pH range of 3 to 10. The pH of the tested solutions was supervised at the beginning of each experiment and hourly thereafter. If necessary, the pH was adjusted to the desired value by adding small amounts of 1M HNO_3_ or 0.1–1.0 M NaOH standard solutions. After adsorption, MNPs were magnetically separated, and the analyte content in the supernatants was separately determined by ICP-OES at 193.759 nm (As), 213.618 nm (P), 224.700 nm (Cu) and 226.502 (Cd) emission wavelengths. The average values of the triplicate measurements were used in all analyses. Arsenic plasma standard solution (Specpure^®,^ As 1000 µg/mL (Alfa Aesar, Madrid, Spain)) was used to prepare calibration solutions. 

The removal efficiency (*R*, %) and adsorption capacity (*q_e_*, mg/g) of the MNPs were calculated as the differences between the amount of studied ions (Cu(II), Cd(II), P(V) and As(III, V)) that were initially added to the system and the amount of the same components that remained at equilibrium in the solutions after the addition of MNPs using the following equations:

$$R\left(\%\right)=\frac{\left(C_{0}-C_{e}\right)}{C_{0}}$$

$$q_{e}\left(\mathrm{mg}/g\right)=\frac{\left(C_{0}-C_{e}\right)\cdot V}{m}$$

where *q_e_* is the amount of analyte adsorbed per mass unit of adsorbent at equilibrium (mg/g); *C*_0_ and *C_e_* are the initial and equilibrium concentrations of the analyte (mg/L), respectively; *V* is the volume of the solution (L); and *m* is the weight of the solid (g).

## 3. Results

Control plants (leaves and roots) and corresponding “hairy” roots of *Artemisia tilesii* were used for biosynthesis of MNPs. Various wormwood sources were used to understanding the mechanism of MNP formation.

### 3.1. ‘Hairy’ Root Extract Preparation and Characterization

**Figure 2:**
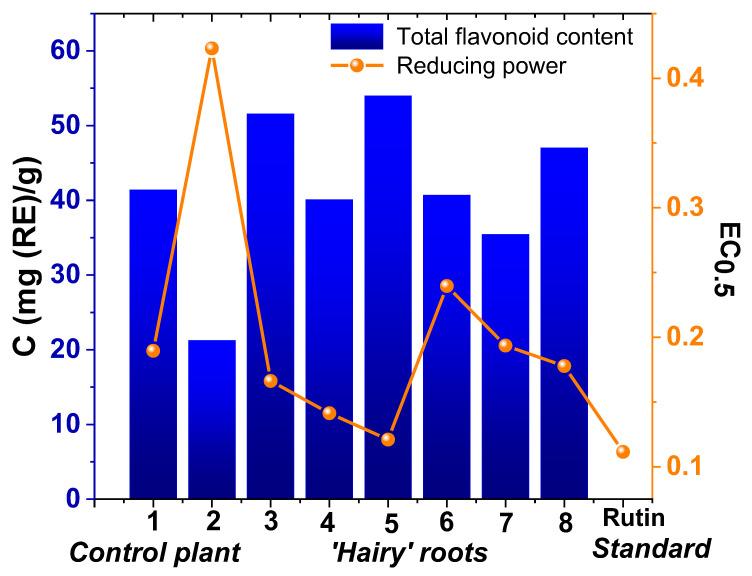
Total flavonoid content and reducing power of ethanolic extracts of *Artemisia tilesii* control plant (leaves and roots) and “hairy” root lines.

The total amount of bioactive compounds and reducing power in ethanolic extracts of *Artemisia tilesii* control plants and “hairy” roots were evaluated as key parameters in the process of biosynthesis of the extracts. These measurements were compared with the reducing activity of rutin solution (1 mg/mL) (Figure 2).

As shown in Figure 2, the reducing power of the obtained extracts was correlated with the corresponding total flavonoid content. Furthermore, total flavonoid content in the extracts obtained from the transgenic roots exceeded, in some cases, the same parameter in the control roots and leaves. In particular, the total flavonoid content in the extracts of transgenic root line No. 5 was higher than in the other extracts, including that of the control plant (54.03 mg (RE)/g). Additionally, the extract of transgenic root line No. 5 had the highest reducing power calculated by the EC_0.5_ value (Figure 2). 

Prior to biosynthesis of MNPs, the bioactive compounds present in the respective ethanolic extracts were identified by ultra-performance liquid chromatography coupled with electrospray ionization time-of-flight mass spectrometry in negative ion mode [40]. The observed peaks of the ion masses were identified for polyphenols corresponding to arginine, quercetin (300.1751 *m*/*z*), sterebin, Sucrose, luteolin-7-b-D-glucopyranosid, isorhamnetin 3-O-glucoside, baicalein-7-O-glucuronide, apigenin-7-O-glucoside, chlorogenic acid, caffeoylquinic acid, caffeoylshiqimic (or dattelic) acid and gallic acid. Furthermore, several peaks in chromatograms can be assigned correspondingly to fragment ions of the unidentified polyphenols.

According to some reports [41,42], the extract ingredients that act as reducing agents, such as sugar, polyphenols, gallic acid and flavones, are among the most effective for the synthesis of metal nanoparticles, including iron oxide. Moreover, the content of these components varied among plants of different species. Thus, the contents of phytochemicals such as flavonoids and phenolic acids that probably participate in the biosynthesis of MNPs were determined in the ethanolic extracts of *Artemisia tilesii* [36]. These components make such extracts potentially suitable for generation of reagents because the polyphenols represent specific ligands for the synthesis and stabilization of MNPs. Study in this direction seems to be promising based on the increase in the content of the named components in the “hairy” roots compared to the original mother plants. These features are associated with the activation of the synthesis of compounds with reducing activity in the cells of ‘hairy’ roots after the incorporation of rol genes of Agrobacterium rhizogenes following the transformation [35].

### 3.2. Synthesis and Characterization of MNPs

In our previous work [36], MNPs were successfully synthesized using a mixture of FeCl_2_/FeSO_4_/CoCl_2_ salts and *Artemisia annua* ‘hairy’ root extracts. In this work, the possibility of MNP synthesis using the same salt mixture and EtOH extracts of the control plants and the six “hairy” root lines of *A. tilesii* was evaluated. 

The addition of *A. tilesii* extracts to the FeCl_2_/FeSO_4_/CoCl_2_ mixture resulted in the solution color changing from colorless to dirty green without precipitation (pH = 2) for several lines (№ 1, № 3, № 5 and № 8). This effect of color change of the mixture solutions correlates with the data on the reducing power of the ethanolic extracts of *Artemisia tilesii* L. (Figure 2). After adding extract № 2 (control roots) to the FeCl_2_/FeSO_4_/CoCl_2_ mixture, the color of the solution did not change. This extract was characterized by the lowest flavonoid content and reducing power. The color of all solutions changed to dark only when the pH was increased to 9.0. The magnet-feeling effect upon separation of the obtained precipitate indicated the formation of MNPs (Supplementary Materials, Figure S1). 

**Figure 3:**
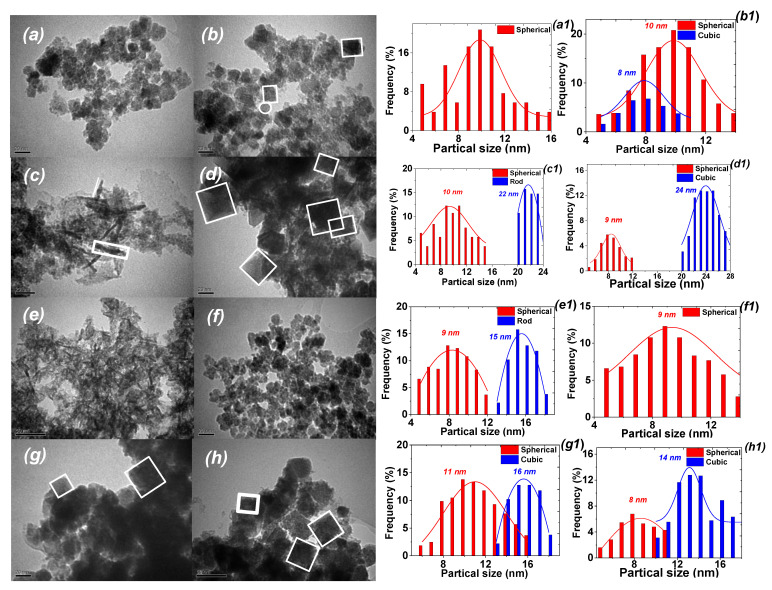
TEM images and particle size distribution of MNPs obtained with EtOH extract of control leaves (**a**,**a1**—№ 1), roots (**b**,**b1**—№ 2) and “hairy” roots of *Artemisia tilesii* (**c**–**h**,**c1**–**h1**—№ 3–8) after addition to the FeCl_3_/FeSO_4_/CoCl_2_ mixture.

The morphology and particle size distribution of the samples prepared by *A. tilesii* ethanolic extracts in the absence of any stabilizer were monitored by TEM (Figure 3).

As shown in Figure 3, particles with different morphologies were formed, predominantly spherical particles with a mean diameter of about 8–11 nm, polyhedral shape (mostly the cube) and nanorods with a mean length of ~15–24 nm and a width of ~2–3 nm. The diameters of the nanoparticles varied from ~8 to ~24 nm depending on the plant material (root lines) used for extract preparation. In the case of MNP-3 used for extract preparation, more nanorods were formed in comparison with MNP-5. The widths of the MNP-3 rods were ~2 nm, similar to the MNP-5 nanorods, but the mean length was shorter, at ~24.5 nm. Spherical nanoparticles with a narrow size distribution were observed for MNP-1 and MNP-2 samples (Figure 3a,b). Figure 3d depicts the cubic shape of MNP-4 with an average size of 24.5 ± 1.6 nm. The MNP-8 sample exhibits a morphology similar to that of MNP-4, with a slightly reduced size covered by ultrasmall MNPs (Figure 3d,h).

**Figure 4:**
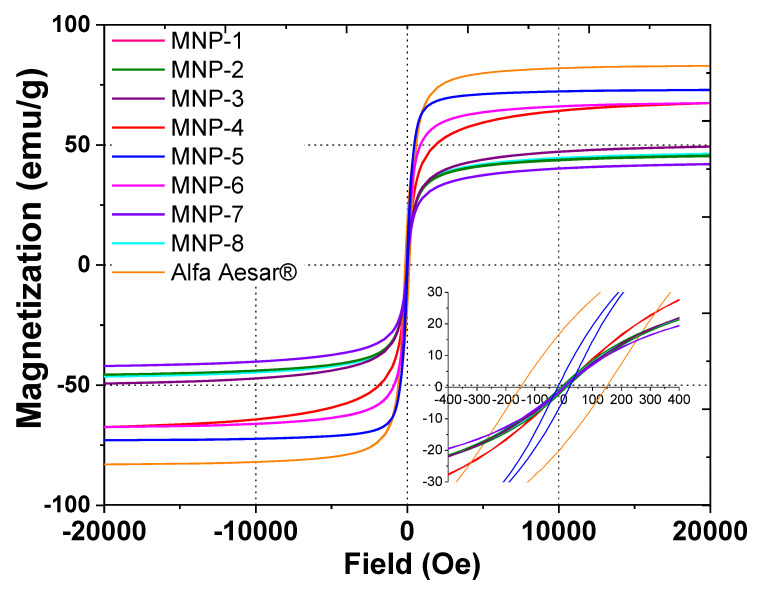
Wide and narrow (insert) magnetic hysteresis loops of biosynthesized MNPs at room temperature.

In the first stage, to compare the magnetic properties of all obtained MNPs samples, magnetization measurements were carried out at ambient temperature until saturation (Figure 4). 

The magnetic properties of the obtained MNPs differed essentially from one another, indicating a significant influence of the nature of the plant extract on the physical characteristics of the resulting solids (Figure 4). Magnetization curves were generated for all samples saturated from 41.7 emu/g to 72.8 emu/g. The saturation magnetization (MS) of the biosynthesized MNPs was lower than that of the bulk (Alfa Aesar^®^, Figure 4) and nanosized (92 emu/g [43]) magnetite standards. A high value of MS is commonly [43] attributed to the stabilization effect of extract components. The highest magnetization saturation was observed for the MNP-5 sample (72.8 emu/g)—about 23% higher than the same characteristic of other MNPs, which may be associated with the high level of reducing power combined with the high flavonoid contents in the extracts used for the synthesis of the MNP-5 sample (Figure 2). In this sense, the flavonoid coating weakens the interaction between the nanoparticles, which reduces the aggregation of MNPs, thus increasing the magnetization saturation. Furthermore, most of the samples had zero coercivity at 300 K, indicating essential superparamagnetic properties of the obtained MNPs [34,43], probably due to the crystallite dimensions of the MNPs, which were in the nanometer size range according to the TEM data. The particle size with high magnetization values, as well as the loss of magnetization after removal of the magnetic field, is an important property of MNPs used for medical and environmental purposes [34]. Only the MNP-5 sample exhibited high hysteresis, indicating the presence of ferromagnetic properties [44]. Magnetic properties of ferrites are directly dependent on the divalent metals present in their composition [45], the synthesis methods and the corresponding preparation conditions. Usually, cobalt ferrite materials have a large coercive field compared to other ferrites [46]. The MNP-5 sample is likely cobalt ferrite. Generally, MNPs prepared by the proposed method were characterized by high saturation magnetization (Table 1) compared to those previously described features of MNPs with non-spherical morphologies [31]. 

To further characterize the nanocrystalline samples, the low-temperature N_2_ adsorption/desorption method was used (Supplementary materials, Figure S2). All the materials were represented by type I and IV isotherms. The type IV isotherms present as a hysteresis loop of H3 type according to the IUPAC classification [47]. Such type I isotherms are characteristic of predominantly microporous materials that consist of agglomerates or approximately uniform particles in regular arrangements, sometimes predominantly spherical in shape [17]. MNP samples with polyhedral morphology (MNP-4, MNP-7 and MNP-8) exhibited predominant mesoporosity and insignificant microporosity. The total mesopore volume is the tendency (Table 1) to decrease for Fe_3_O_4_ (where the predominant mesopore volume is in pores with diameters of 1.6–2.1 nm for MNP-4 and MNP-7) (Supplementary material, Figure S3). Thus, the use of ‘hairy’ root extracts in MNP synthesis resulted in increased porosity and specific surface area formation (S_BET_).

**Figure 5:**
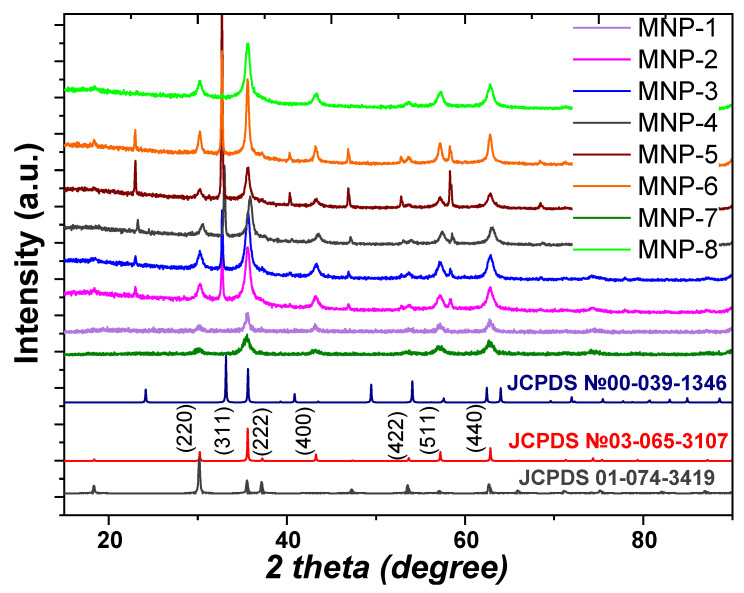
Powder XRD patterns of obtained MNPs using ethanolic extract of *Artemisia tilesii* and reference materials (magnetite, maghemite and cobalt ferrite).

A diffraction technique, namely the X-ray powder diffraction method (Figure 5), was applied to identify the phases present in the prepared magnetic materials. Additionally, the corresponding patterns of magnetite (JCPDS Card No. 03-065-3107), maghemite (JCPDS Card No. 00-039-1346) and CoFe_2_O_4_ (JCPDS Card No. 01-074-3419) are shown because maghemite occurs as a fully oxidized form of magnetite and resembles magnetite in structure and magnetic properties.

In the XRD patterns (Figure 5), characteristic peaks were observed at 2θ = 30.1°, 35.5°, 43.1°, 53.4°, 57°, 63.1° and 74.9°, which were related to the corresponding indices of (220), (311), (222), (400), (422), (511), (440) and (533), respectively. The experimental data mentioned above are in accordance with JCPDS Card No. 03-065-3107 for magnetite (Fm3m) with a face-centered cubic spinel crystalline structure. Moreover, some additional peaks in several samples indicate the presence of crystalline impurity. The sharp peaks around 2θ = 22.9°, 32.5° and 58.2° match the pattern of NH_4_Cl (JCPDS Card No. 01-077-2352), i.e., residuals of ammonium chloride formed during the synthesis using Cl^-^ and NH_4_OH, which were not removed during the proposed washing. The intensity of these peaks decreased after multiple washings of the samples with distilled water, but this treatment led to sample loss. On the other hand, the peaks shown in Figure 5 indicate that the magnetite phase was clearly detected in the XRD pattern without the maghemite phase as impurities. The XRD patterns of most obtained MNPs samples exhibits the appearance of a “halo” at 2θ = 20–30° 2 theta, which was generally related to the presence of an amorphous phase. This phenomenon was probably the result of the considerable amount of non-crystalline phase in the composition of MNPs, i.e., the nanoparticles were covered by bioactive organic components of the extract, which were detected as an amorphous phase. The average crystal sizes for the magnetic samples were determined from the XRD line broadening of the most intense (311) diffraction peak. The experimental data show that the modification of the reaction mixture by the use of various extracts led to changes in the characteristics of the obtained materials, such as porous structure, the size of the particles, crystallinity and magnetic properties. 

Chemical analysis of the obtained samples was performed using a combination of EDX spectroscopy and ICP-OES techniques (Table 2). EDX spectra (Supplementary Materials, Figure S5) demonstrated that iron, cobalt and oxygen were the main elements in the MNPs. EDX analysis revealed the presence of residual chlorine and sulfur as impurities. Additionally, the EDX data depict carbon and nitrogen signals, most likely due to the presence of bioactive compounds and/or NH4Cl on the surface of MNPs. Although the EDX patterns are similar to the results acquired in our previous report [36], slight differences in the element intensities were found, which are likely related to variations in the synthesis conditions. 

According to the ICP-OES results (Table 2), the concentration ratio (at. %) of Fe/Co in the samples was close to the assumed theoretical values and showed an increasing content of substitution of Fe by Co as follows: 43.88/5.01, 42.52/6.11, 44.09/3.52, 46.09/2.12 and 45.09/3.01 for the MNP-1, MNP-2, MNP-3, MNP-5 and MNP-6 samples, respectively. The chemical analysis performed for the MNP-4, MNP-7 and MNP-8 samples showed the absence of Co, which means that this atom was not incorporated in the structure of these MNPs. The results indicate that these samples are pristine magnetite. These results were confirmed by the existence of magnetic hysteresis loops in the mentioned samples.

**Figure 6:**
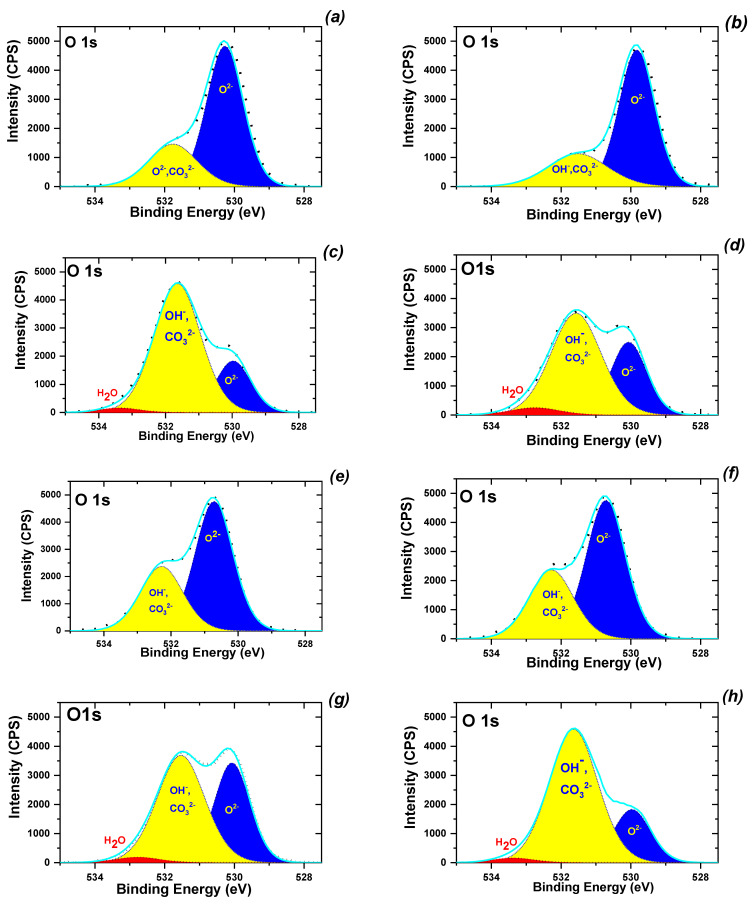
XPS spectra of O1s speciation on the surfaces of MNP-1 (**a**), MNP-2 (**b**), MNP-3 (**c**), MNP-4 (**d**), MNP-5 (**e**), MNP-6 (**f**), MNP-7 (**g**) and MNP-8 (**h**).

In addition, the detailed chemical state of the surface components in the as-biosynthesized magnetic samples was observed by XPS analysis. The XPS survey spectra (not shown) of the obtained nanoparticles show strong bands associated with iron at 724 eV (Fe2p_1/2_), 709 eV (Fe2p_3/2_) and 60 eV (Fe3p); carbon at 285 eV (C 1s); nitrogen at 398,8 eV (N 1s); oxygen at 515 eV (O 1s); and cobalt at 539 eV (Co 1s). The peak at 539 eV is typical of cobalt. This band was present only in the spectra of the MNP-1, MNP-2, MNP-5 and MNP-6 samples. The presence of a band at 285 eV (≈7–17 at.%) in all studied samples was probably due to carbon adsorption on the nanoparticles or impurities of other origins. Multiple splitting of the Fe 2p spectra of MNPs is revealed in Figure S4. The high-resolution spectrum of Fe2p shows, in addition to a strong, narrow band at 709.5 eV (Fe2p_3/2_) and a weaker band at 722.6 eV (Fe2p_1/2_), other satellite peaks, including one located about 8 eV higher than the main Fe2p_3/2_ peak and another 8.4 eV higher than the weakest Fe2p1/2 band. The surface oxygen speciations of the obtained MNPs from O1s XPS spectra were estimated as illustrated in Figure 6.

It is clear that the O1s spectra of the prepared MNPs differ considerably from one another, indicating that all samples have different ratios of various oxygen chemical states depending on the nature of the extracts (Figure 6). The two peaks at 529.88 and 532.36 eV in the O1s spectrum do not take into account the binding energies corresponding to the Fe−O and Co−O bonds as expected. The O(1s) spectra of each adsorbent consisted of overlapping peaks of oxygen in the oxide (O^2−^), hydroxyl groups (OH^−^), carbonate (CO_3_^2−^), adsorbed water (H_2_O) and other oxygen-containing molecules. As shown in Figure 6a,b, if the sample contained a considerable amount of Co (for example, in the MNP-1, MNP-2 and MNP-5 samples), the concentration of OH^-^ species on the surface was lower than the concentration of pristine magnetite (MNP-3, MNP-4 and MNP-8 samples). Thus, the use of ‘hairy’ root extract in the synthesis (Table 1) resulted in a significant increase in OH- species on the surface of samples. 

**Figure 7:**
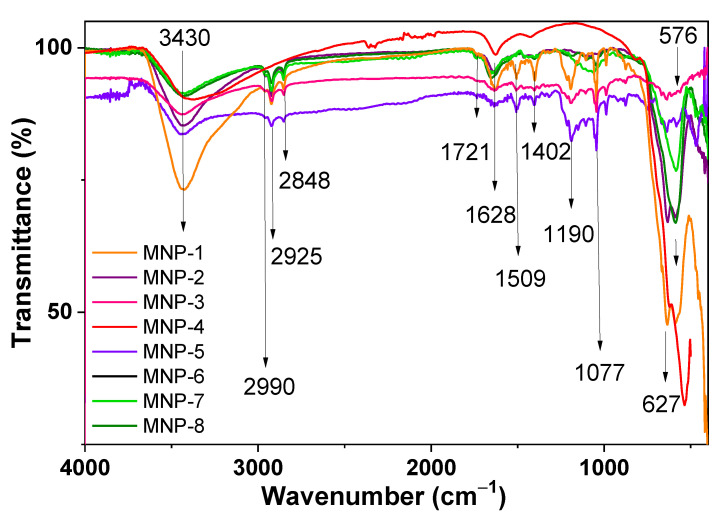
FTIR spectra of various as-prepared MNPs.

To verify the evolution of the surface functional groups of the obtained magnetic materials, the FTIR spectroscopy technique was used. As shown in Figure 7, the FTIR spectra of iron oxides exhibited strong bands in the low-frequency region (1000–300 cm^−1^). The appearance of an Fe-O bond at about 576 cm^−1^ confirms the formation of compounds based on iron oxides. However, we observed that the infrared absorption near this peak presented some additional features, such as shoulders near 590 and 630 cm^−1^. Maghemite is known to exhibit IR absorption at 630 cm^−1^ [15]. Therefore, the partial oxidation of magnetite to maghemite (γ-Fe_2_O_3_) is a possible explanation for the observation of the IR absorption near 630 cm^−1^. The oxidation of Fe(II) to Fe(III) in magnetite was also observed in other studies, for example, by Chourpa et al. [48]. The IR spectra of CoFe_2_O_4_ nanoparticles prepared according to different procedures usually show two absorption bands—at 389 cm^−1^ and ∼586–590 cm^−1^ [28]. Thus, a very strong absorption band of the IR spectra of the obtained MNPs centered at 591 cm^−1^ was assigned to a vibrational mode with contributions from magnetite and Co-ferrite phases in the nanoparticles. For the MNP-1, MNP-2, MNP-3 and MNP-5 samples, the two peaks observed in this region (at 576 cm^−1^ and 590 cm^−1^) were assigned to the Fe-O and Co-O vibration from the CoFe_2_O_4_. In the region of 2800–3000 cm^−1^, absorption bands characteristic of valence vibrations of C-H bonds (sp^3^, sp^2^ and sp hybridization) were observed, indicating the presence of organic motifs in most samples. The strong and broad band at ~3430 cm^−1^ is attributed to the ν_s_(OH) stretching vibration of water-binding water molecules. Moreover, stretching vibration peaks of water appeared at 1630 cm^−1^. The absorptions centered at 1509 cm^−1^ (ν_as_(COO)) present high intensities in the IR spectra. In addition, the peaks at 1721 cm^−1^ were associated with oscillations of the C=O bonds in all samples. As expected, the above-mentioned stretching vibration peaks of organic oxygen-containing functional groups disappeared in the spectrum of the MNP-1, MNP-2, MNP-3 and MNP-5 samples, i.e., the organic bioactive compounds had been originated by the plant extracts. In the FTIR spectra of all samples, the most intense absorption band was observed in the region of 1050–1080 cm^−1^, which indicates the presence of ν(C-O) bonds of organic fragments. Moreover, according to the FTIR spectra, the magnetic core (Fe_3_O_4_ or CoFe_2_O_4_) was covered by the organic compounds of the plant extracts, which is consistent with the EDX data.

As reported in [34], the amount of OH- is a key factor for the adsorption of pollutants. The atomic ratio of Fe/Co and organic components of extracts on the surface of MNPs considerably influences on the chemical composition of these adsorbents and their adsorption performance with respect to cationic and anionic species.

### 3.3. Adsorption Performance toward Cation and Anion Forms of Toxicants

In order to examine the adsorption performance of the new nanoparticles, the sorption of Cd(II), Cu(II) and As(III, V), P(V) ions from individual and mixture aqueous solutions was investigated in batch experiments.

**Figure 8:**
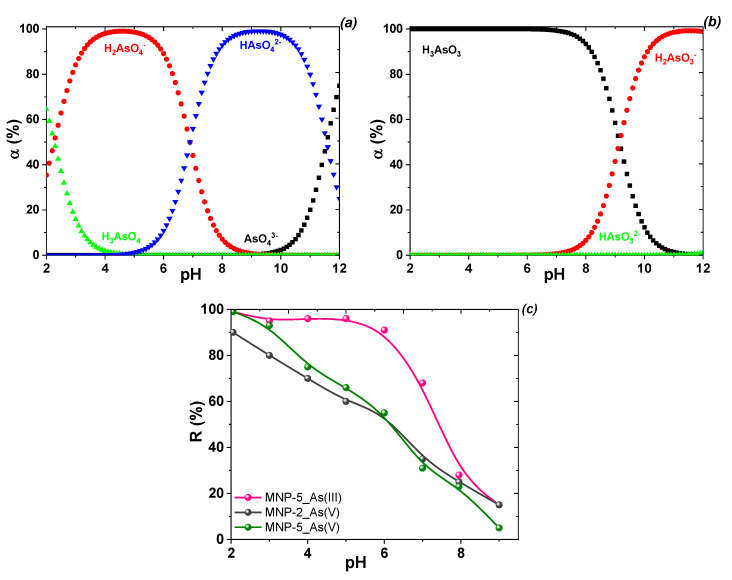
Effect of pH on arsenate (**a**) and arsenite (**b**) speciations in aqueous solution and their removal by selected samples (MNP-2 and MNP-5) (**c**) (conditions: C_0_(As(V)), 2 mg/L; treated volume, 10 mL; adsorbent dosage: 30 mg; treatment time, 2 h at room temperature).

***Anion removal.*** In the environment, arsenic occurs mainly as an inorganic species, including as arsenites and arsenates in natural water [5]. First, the optimum working pH and speciation of arsenate anions in the coagulation adsorption process were evaluated in this study. The speciation of arsenate anions depends on the pH of the medium (Figure 8a,b). In aqueous medium, As(V) species are presented as H_2_AsO_4_^−^ and HAsO_4_^2−^ under near-neutral conditions (pH 6–8) and AsO_4_^3−^ at pH > 9, although H_2_AsO_4_^−^ species are dominant at pH 4−5. To investigate the effect of pH for As(III, V), adsorption was used with selected MNPs in the pH range of 2–9 (Figure 8c). As the nature of the sorption (the higher the pH, the lower the sorption) was preserved, studies on the dependence of adsorption on pH value of P(V) were not performed because phosphorous and arsenic elements belong to five groups of the periodic table with similar chemical properties. Both arsenate and phosphate ions have a tetrahedral architecture with close radii and very similar protoltic and adsorption properties [11,49].

The adsorption of arsenate and arsenite onto selected MNPs was strongly pH-dependent (Figure 8c). For arsenate and arsenite anions, the adsorption percentage was obviously enhanced as pH decreased. The influence of pH on the adsorption of As(III) and As(V) differed, as the interaction between the two arsenic species and the MNP surface may change depending on the variation in pH. pH affects both the As(V) species present in aqueous media and the surface charge and dissociation of hydroxyl groups from MNPs because increasing pH decreases the proportion of positively charged surface sites on MNPs. As As(V) is in the anionic form under most pH conditions, arsenate uptake decreases with increasing pH. In turn, absorption of arsenite does not change significantly, as it is mostly in molecular form at pH < 9.0 (Figure 8b). At pH 5 and above, the total negative surface charges of the material increase, and the number and strength of interphase hydrogen bonds gradually decrease. In contrast, in the alkaline pH range, the increased OH^−^ in the solution suppresses the interaction between anionic As species and hydroxyl groups of MNPs, triggering diminished adsorption capacity. Thus, whereas a decrease in anion uptake is observed at pH values above 5, the use of dissolved adsorbents to remove these analytes with acceptable values seems useful up to pH 8. The adsorption of As(V) on the studied MNPs decreases in the pH range of 4–7, which favors the ionic interaction with the H_2_AsO_4_^−^ anions.

The release of iron ions into solution in anion adsorption experiments was occasionally measured. The release of Fe^3+^ ions into solution ranged from 0.0 to 0.08 ppm at pH 6.5 to 8.0. On the other hand, at pH 3, iron release increased significantly, with values of 52, 109, 31, 3 and 84 ppm for the MNP-1, MNP-2, MNP-3, MNP-5 and MNP-7 samples, respectively.

**Figure 9:**
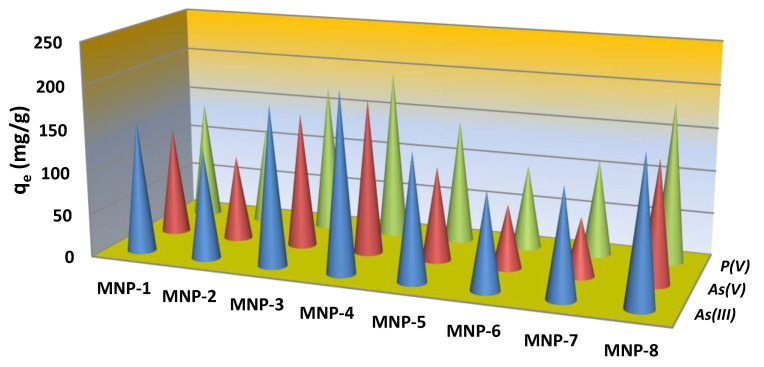
Adsorption performance of the obtained MNPs to phosphate, arsenate and arsenite anions (conditions: adsorbent dose, 20 mg/L; pH 6.7 ± 0.2; initial concentration of all anions, 150 mg/L; ambient temperature).

The maximum adsorption capacity of all obtained magnetic products by adsorption of several anions (phosphate, arsenate and arsenite) was tested. Figure 9 shows the adsorption performance of the eight magnetic solids to oxyanions under the same experimental conditions. To estimate the adsorption capacity of the materials at the pH of drinking water (6.5–8.5), experiments were conducted at an adjusted pH of 6.7 (±0.2). The initial concentration of all anions was sufficient (200 ± 15 mg/L) to estimate adsorption close to the saturation of adsorption sites, which is often reflected in equilibrium isotherms by a plateau.

Among all the studied samples, MNP-4 exhibited the best anion removal performance (Figure 9). Its phosphate and arsenate adsorption capacity was the highest, followed by MNP-3 and MNP-8. The non-doped by Co magnetite-based materials with the same efficiency adsorbed all studied anions, i.e., AsO_4_^3−^, AsO_3_^3−^ and PO_4_^3−^. The Co-containing samples with a minimal amount of Fe (MNP-6 and MNP-2) achieved the poorest performance with AsO_4_^3−^ and PO_4_^3−^. Only one sample based on non-purely magnetite-based compounds (MNP-1) removed AsO_4_^3−^ and PO_4_^3−^. Additionally, high PO_4_^3−^ concentrations (1 mg/g) had a significant effect on arsenic adsorption in multicomponent solution. The effect of phosphate on arsenate and arsenite adsorption was negligible only when the concentration of PO_4_^3−^ (0.1 mg/g) was less than the concentration of AsO_4_^3−^.

For the three tested anions in the individual analyte systems, the adsorption capacity of the studied MNPs was observed to follow the order of As(III) > As(V) > P(V). Notably, the adsorption performance of MNP-4 to As(V) was outstanding compared to analogs synthesized among magnetic materials described in the literature (Table 3).

To evaluate the applicability prospects of these materials in water treatment, many other experiments should be performed to study the effect of competing ions and performance under dynamic adsorption conditions. Based on the experimental data, it can be concluded that the removal efficiency of arsenate and phosphate under the experimental conditions described above is competitive using the materials employed in the present study. Such efficiency is usually provided by adsorbents with sufficiently high adsorption affinity for these anions.

**Figure 10:**
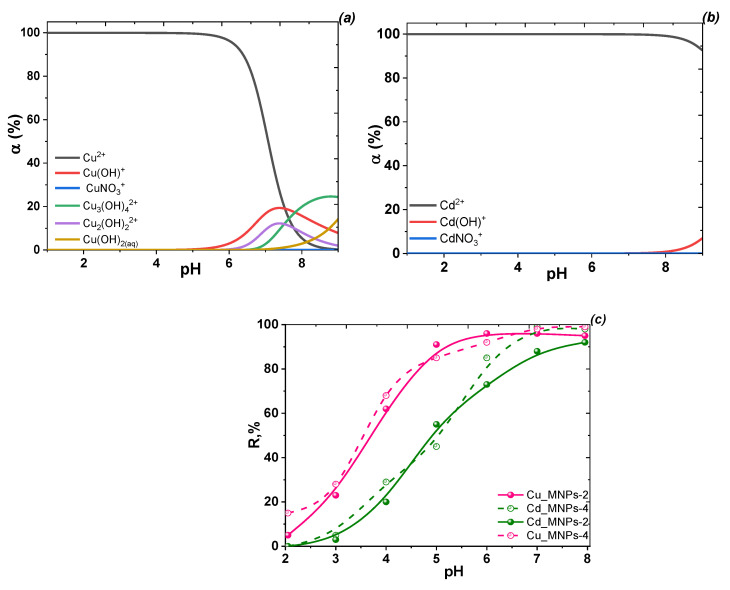
Effect of pH on the distribution forms of Cu(II) (**a**) and Cd(II) (**b**) in aqueous solution and its removal by MNPs (**c**) (Conditions: C(Cu(II)) = C(Cd(II)) = 0.001 M (64.5 mg/L and 236.4 mg/L, respectively), adsorbent dose 50 mg, volume 15 mL, contact time 1 h, ambient temperature (20 ± 2 °C)).

***Cation removal.*** Copper(II) in an aqueous solution (Cu(NO_3_)_2_ salt as precursor) exists as Cu(II) ions only in the acidic region (up to pH ~5.0) and as hydroxocomplexes with varying chemical compositions in neutral and alkaline media (Figure 10a). According to the diagram of the distribution forms of Cd(II) ions in the aqueous solution of Cd(NO_3_)_2_ (Figure 10b), Cd(II) ions can be adsorbed on the synthesized MNPs in a single form of Cd(II) up to pH~6.7 (close to the native pH of environmental water). The effect of pH in the range of 3–9 on the adsorption of the metal ions by a series of MNPs (based on magnetite and ferrite) was studied (Figure 10c).

The effect of pH on Cu(II) and Cd(II) ion adsorption on the studied solids (Figure 10c) shows that the higher the pH of the initial solution, the better the adsorption on MNPs. This can be explained by the weakly acidic cationic nature of MNPs due to amphoteric properties. Generally, the surface Fe−OH sites of Fe_3_O_4_ can undergo protonation (Fe−OH + H^+^ ↔ Fe−OH_2_^+^) and deprotonation (Fe−OH ↔ Fe−O−) reactions under acidic and alkaline conditions, respectively. This phenomenon also occurs because the organic fragments have weakly acidic acid residues that dissociate at pH values close to neutral. As experiments revealed that the highest adsorption occurs at pH = 5.5–6.5, whereas the formation of metal hydroxycomplexes had not yet occurred, further copper cation sorption studies were performed at pH near 6.5. 

**Figure 11:**
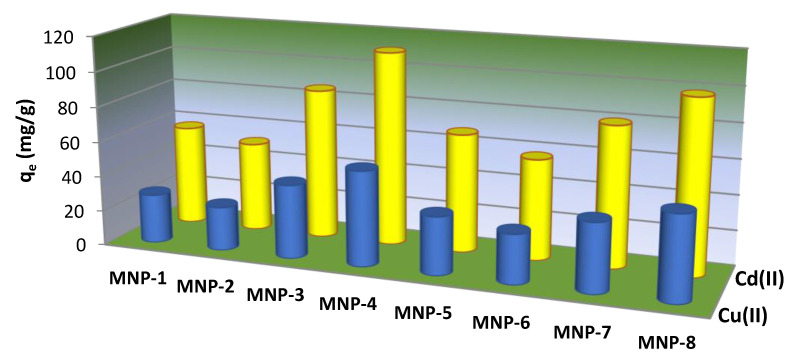
Comparison of the adsorption activity of the MNP samples during the adsorption of the studied cations from individual component solutions at pH 6.7.

The maximum adsorption capacities of the obtained MNPs toward Cu(II) and Cd(II) ions were investigated using initial concentrations of 2 mg/L at a pH value close to 6.7 (Figure 11). The other adsorption parameters were previously defined above.

As shown in Figure 11, the obtained MNPs exhibited various removal efficiencies towards the studied metal ions. The adsorption capacity for Cu(II) ions is about 28.2 mg/g (0.44 mmol/g), 25.4 mg/g (0.39 mmol/g), 42.3 mg/g (0.66 mmol/g), 54.2 mg/g (0.85 mmol/g), 33.4 mg/g (0.52 mmol/g), 28.5 mg/g (0.45 mmol/g), 39.0 mg/g (0.61 mmol/g) and 48.3 mg/g (0.76 mmol/g) for the MNP-1, MNP-2, MNP-3, MNP-4, MNP-5, MNP-6, MNP-7 and MNP-8 samples, respectively. According to the literature [7], the interaction usually occurs as a result of the complexation of one target metal ion with organic groups (ligands). The observed differences in maximum capacities of the studied metal ions are most likely owing to their differing affinities to interact with bioactive organic groups on the surface of magnetic adsorbents according to the stability constant of the complexes of each of the tested metal ions. The amount of removed Cd(II) cations was ~1.7–1.8 times higher than that of Cu(II), which was also supported by the data from adsorption measurement (Figure 11). These data indicate that Cu(II) and Cd(II) ions bind to similar types of adsorption centers. The sorption capacity of the samples correlates with morphology (Figure 3) and does not depend on porosity (Table 1). The highest adsorption capacity was observed using MNPs with non-spherical forms. The lowest sorption performance was observed for the samples obtained with the control plant extracts. This effect is likely the result of the low content of bioactive organic components, which play a dominant role in the affinity of MNPs toward metal ions. 

Experimental data (Figure 11) show that the adsorption capacity of Cd(II) ions was equal for most of the studied samples. Thus, the used biosynthesized MNPs had bioactive shell-like layers [36], which acted as ligands for target metal ions. Furthermore, the sorption capacity of the MNPs obtained in this study is close to the adsorption capacity of modified materials [7,12]. 

The adsorption mechanism for these materials was not specifically evaluated. However, the high adsorption capacity toward both adsorbates on obtained MNPs could be explained by the strong specific interaction of the surface groups of magnetite or ferrite (≡Fe-OH or ≡Co-OH) with heavy metal ions, possibly involving a complexation mechanism [7,12] and a simple surface-exchange reaction, which can be simply expressed in general form as

≡FeO-H + Cu^2+^ → ≡FeO-Cu^+^ + H^+^

This reaction occurs without byproducts according to ICP-OES data. Thus, the obtained materials offer a promising alternative for the uptake of heavy metals during water purification.

### 3.4. Adsorptive Performance of the MNPs to Cations and Anions as a Function of (Phase, Element) Speciation, Surface Chemistry and Porous Properties

**Figure 12:**
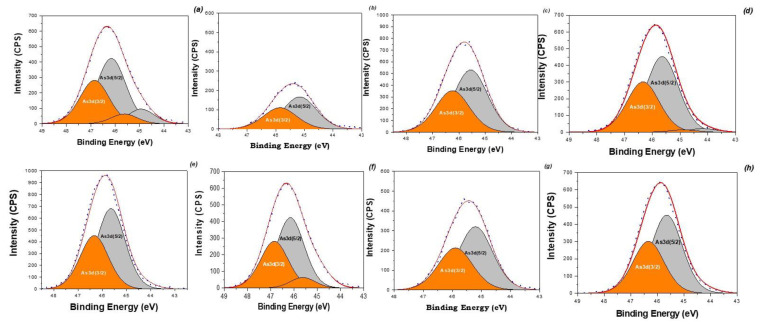
XPS patterns of As 3d binding energy of the MNPs (MNP-1 (**a**), MNP-2 (**b**), MNP-3 (**c**), MNP-4 (**d**), MNP-5 (**e**), MNP-6 (**f**), MNP-7 (**g**) and MNP-8 (**h**)) saturated with As(V) at 25 °C.

Recently [49,58], it was reported that the removal of oxy-type anions by iron oxides or hydroxides was dependent on the ability of the surface hydroxyl groups (-OH) to form outer-sphere and inner-sphere complexes with metal ions. In this study, the interactions between the obtained MNPs and As(V) were verified by means of XPS, as shown in Figure 12.

Figure 12 shows that the As 3d level after adsorption is divided into two spectra at 44.54 and 45.56 eV, which are assigned to As(III) and As(V), respectively. This duplet peak of As 3d suggests that a fraction of As(V) was reduced to As(III) during adsorption and chemical bonding with Fe. The Fe(II) of MNPs had a significant reductive capacity under ambient conditions and therefore reduced As(V) to As(III) adsorbed on MNPs through redox transformation. Therefore, octahedral Fe(II) and Fe(III) ions can oxidize and reduce reversibly during the reaction with adsorbed anions, such as AsO_4_^3−^ and AsO_3_^3−^, without affecting the spinel structure. This inverse spinel structure of magnetite toward As(V) was previously reported in [59]. The data also suggest that iron oxide’s OH groups play a substantial role in As(V) sorption through ion exchange between OH− and As(V), as expressed by following equation:

≡FeO-H + H_2_AsO_4_^−^ → ≡FeO-(AsO_3_)OH^−^ + H_2_O

As(III) has a strong attraction to the iron oxide surface and forms inner-sphere bidentate binuclear and mononuclear complexes of As(III) with Fe(III) in the Fe_3_O_4_.

The changes of O1s binding energy on the MNPs surface increased by 532.39, 532.26 and 532.35 eV after adsorbing As(V), indicating the decrease in electron density in the O1s shell, confirming the mechanism of complex formation between hydroxyl groups and pollutants of the oxyanion type [60]. Additionally, the iron oxide’s hydroxyl groups (OH−) play a substantial role in As sorption through ion exchange between OH− and As [60,61]. As(III) has a strong attraction to the iron oxide surface and forms inner-sphere bidentate binuclear and mononuclear complexes of As(III) with Fe(III) on the surface of MNPs at pH ≥ 7 owing to the electrostatic repulsive force between these two negatively charged surfaces (As oxyanions and magnetite).

On the other hand, outer-sphere bidentate binuclear complexes can be formed between As(V) and FeOH_2_^+^ of the Fe_3_O_4_ surface at pH < 7. After the formation of outer-sphere complexes, inner-sphere complexes may also be developed because of the coulombic interaction between the Fe_3_O_4_ surface and arsenate oxyanions by ligand exchange [49].

Although the initial pH of the solution was 5.0, the final pH after adsorption was close to neutral, so the formation of both outer-sphere and inner-sphere complexes in the solid phase is possible. In summary, on the basis of all of the aforementioned findings and explanations, it can be concluded that adsorption occurred as a result of the partial reduction of As(V) to As(III) through the formation of inner-sphere and outer-sphere complexes, which was possible due to ion exchange and the interaction between As and (hydro)oxides of the Fe_3_O_4_ surfaces.

### 3.5. Safety Evaluation of MNPs through Phytotoxicity Studies

An accurate investigation of toxicity of the obtained MNPs is recommended, as part of these adsorbents can enter water sources after adsorption of various pollutants [62]. Traditionally, the in vivo toxic effects of nanomaterials are revealed via animal studies [63]. Although extremely informative, animal studies are expensive and time-consuming and are therefore not suitable for the systematic study of the multitude of potential variables of nanomaterials. In vitro studies with different plants cannot substitute studies using animals. Furthermore, this approach can help to reveal structure–activity relationships and suggests that nanomaterials are not likely to exhibit toxic activity in vivo. 

**Figure 13:**
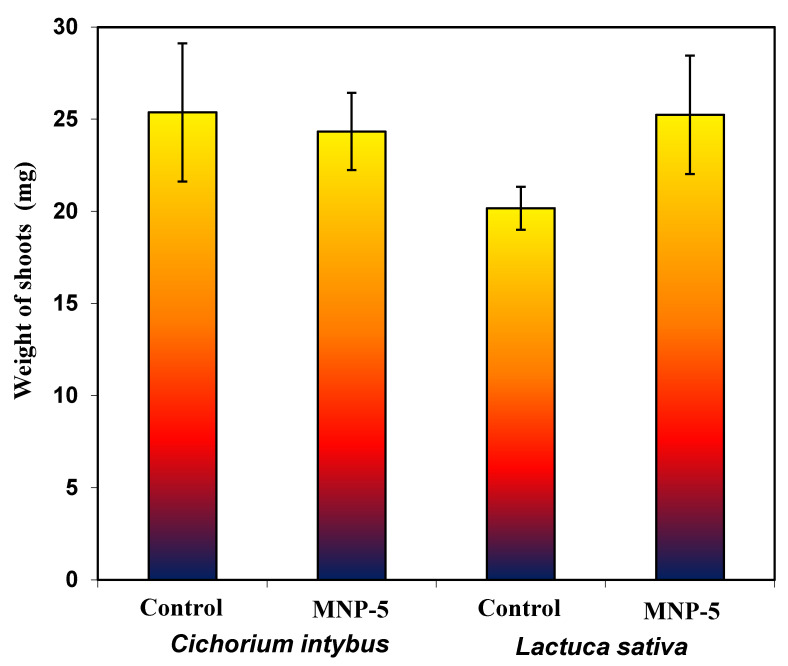
The effect of MNPs on the shoot weight of *Cichorium intybus* L. and *Lactuca sativa* L. (control plants and pretreated with the MNP-5 sample).

Phytotoxicity studies using *Cichorium intybus* L. (“*Palla Rossa*”) and *Lactuca sativa* L. (“*Izumrudny*”) plants were carried out to determine the probable inhibitory potential of MNPs. These plants are characterized by fast and active seed germination (first seedlings appear in 3–5 days), and it is believed that lettuce seeds are sensitive to metal toxicity, owing to their small size [64]. The effect of MNP-5 on the weight of germinated seeds is shown in Figure S5. Lettuce and chicory seedlings were incubated at 22 °C for 12 days after exposure to MNP-5 suspensions. After this period of time, the growth of normal green plants was observed from both chicory and lettuce seeds. The weights of the shoots of the control and pretreated samples are depicted in Figure 13.

The percentage of germinated chicory and lettuce seeds in the control was 64% and 74%, respectively. The seed treatment did not inhibit the germination process, as the percentage of germinated seeds was 69% and 72% for chicory and lettuce, respectively, in the experimental variant. There were also no statistical differences in the weight of pretreated and control seedlings, as shown in the Figure 13. 

These preliminary results demonstrate that biosynthesized MNPs do not have an inhibitory effect compared to other oxide adsorbents, including nanoparticles [64]. However, even with such results, additional research directed at water local organisms is needed.

## 4. Conclusions

In this study, an efficient and environmentally friendly method of “green” synthesis of MNPs was proposed using extracts of *Artemisia tilesii* L. “hairy” roots. The influence of various synthesis parameters on the morphology of the magnetic nanoparticles was been determined. TEM data revealed that the particles have varied morphologies (spherical, cubic and nanorod). The size of the nanoparticles can be tuned from ~8 to ~24 nm by changing the lines of the extracted ‘hairy’ roots. The obtained MNPs were materials with a spinel structure (magnetite or ferrite). According to FTIR data, synthesized MNPs have a magnetic core and a shell formed by bioactive molecules. These results constitute the first successful report of the use of “hairy” root extracts for nanoparticle biosynthesis of Fe_3_O_4_ and CoFe_2_O_4_. The focus of the present study was to understand the interaction between heavy metals, As(III, V) and P(V) ions with two well-known classes of potential adsorbents, i.e., magnetite or ferrite, in the important pH for environmental remediation. The best adsorption was demonstrated by materials based on Fe_3_O_4_ of low crystallinity, which had a polyhedral morphology and contained a large number of surface functional groups on the exposed surfaces. These Fe_3_O_4_ samples with polyhedral shapes were better adsorbents of heavy metal ions. The synthesized MNPs effectively removed metal ions from the aqueous medium with an acceptable adsorption capacity, making them potentially useful for environmental remediation. Phytotoxicity analysis evidenced that biosynthesized MNPs has no inhibitory effect on *Lactuca sativa* L. and *Cichorium intybus* L. seeds. These MNPs have many potential applications for adsorption, catalysis, etc. Finally, the proposed synthesis method is facile, shape-controllable and versatile for selective and exclusive loading of a series of other nanoparticles. 

## Figures and Tables

**Table 1:** Summary of main physical characteristics of the obtained MNPs.

Sample	d^1^, nm	d^2^*,* nm		S_BET_, m^2^/g	d_por_, nm/ V_tot_, cm^3^/g	M_S_, emu/g	H_C_, Oe
		Spherical	Other Shapes				
* Alfa Aesar^®^	-	50–100	–	20–50	-	83.0	18.53
MNP-1	12.1	10.5 ± 3.0	–	23	0.4/0.42	42.1	0.18
MNP-2	10.21	10.1 ± 3.2	8.5 ± 2.0	35	0.3/0.61	45.8	0.85
MNP-3	16.11	10.0 ± 2.9	22.1 ± 1.0	51	0.6/0.84	49.4	0.02
MNP-4	17.23	9.5 ± 1.5	24.5 ± 1.6	105	2.04/0.61	67.4	0.29
MNP-5	10.6	9.0 ± 2.0	15.2 ± 1.9	90	1.0/0.42	72.9	3.72
MNP-6	16.11	9.1 ± 1.9	–	85	1.6/0.74	67.4	3.70
MNP-7	17.23	11.2 ± 3.2	16.0 ± 2.2	115	2.12/0.41	42.0	0.02
MNP-8	12.6	8.1 ± 2.1	14.1 ± 2.5	90	1.8/0.32	46.6	0.27

**Notes**. * Reference material of magnetite. d^1^—crystallite size evaluated based on XRD data [37]; d^2^—particles sizes evaluated by TEM method; S_BET_—specific surface area calculated by BET method; V_tot_ and d_por_—total volume and diameter of pores, respectively, evaluated by BJH method.

**Table 2:** Synthesis conditions and chemical composition in the resulting samples.

Sample	Ratio in Reaction Mixture Fe^3+^/Fe^2+^/Co^2+^/Extract	Chemical Composition in Final Solids				Ratio Fe^n+^/Co^n+^	Composition
		Fe, %	Co, %	C, %	H, %		
MNP-1	2.0 mL (5.4%)/2.0 mL (1.9%)/0.2 mL (2%)/1.0 mL	12.86	66.8	0.19	0.33	1:2.07	CoFe_2_O_4_
MNP-2		15.91	57.47	0.13	0.50	1:1.44	CoFe_2_O_4_
MNP-3		55.55	-	2.52	1.40	-	Fe_3_O_4_
MNP-4		61.02	-	0.35	0.62	-	Fe_3_O_4_
MNP-5		20.77	40.07		0.63	1.30:1	CoFe_2_O_4_
MNP-6		25.81	37.67		-	1.69:1	CoFe_2_O_4_
MNP-7		61.41	-	2.89	0.70	-	Fe_3_O_4_
MNP-8		79.96	-	-	0.44	-	Fe_3_O_4_

**Note.** *n* = 2,3.

**Table 3:** Comparison of adsorption capacity toward As(III, V) with some magnetic adsorbents.

Adsorbent	Adsorption Conditions	Adsorption Capacity (mg/g)		Reference
		As(III)	As(V)	
Fe^0^/C	pH 7.0	18.9	12.02	[50]
Iron oxide-loaded slag	pH 10 (As(III)), pH 2.5 (As(V))	2.9–30	18.8–78.5	[51]
Fe(III)/lysine-N^α^,N^α^-diacetic acid	pH 9 (As(III)), pH 3.5 (As(V))	62.93	55.44	[52]
Bead cellulose-loaded iron-oxy-OH	pH 7.0	99.6	33.2	[53]
Fe(OH)_3_-coated Al_2_O_3_	pH 6.6–7.2	7.64	36.64	[54]
Iron-modified activated carbon	pH 8 (As(III)), pH 6.0 As(V))	39.2	51.3	[55]
Iron oxide-doped chitosan composite	pH 7.0	22.47	16.1	[56]
Natural goethite hematite magnetite	pH 7.5	10.1	12.1	[57]
	pH 7.3	10	31.3	
	pH 6.5	10.0	12.0	
Magnetite and cobalt ferrite	pH 6.7	2.9–30	18.8–78.5	This work

## Data Availability

Not applicable.

## Supplementary Materials

The following supporting information can be downloaded at: https://www.mdpi.com/article/10.3390/nano12234231/s1, Figure S1: Biosynthesized MNPs using EtOH extract of *Artemisia tilessia* (control plants (leaves and roots) and *“hairy*” roots) and a FeCl_3_/FeSO_4_/CoCl_2_ mixture at pH 9; Figure S2: N_2_ ad/desorption isotherms and pore size distribution curves for all as-prepared MNP samples; Figure S3: High-resolution XPS *Fe2p* spectra of as-biosynthesized magnetic samples; Figure S4: EDX spectra combined with microanalysis of MNP-1, MNP-2, MNP-3, MNP-4, MNP-5, MNP-6, MNP-7 and MNP-8 samples. Figure S5: *Cichorium intybus* L. and *Lactuca sativa* L. control plants and plants pretreated by MNPs.
