# Supplementary material for: Morphology-Controlled Green Synthesis of Magnetic Nanoparticles Using Extracts of ‘Hairy’ Roots: Environmental Application and Toxicity Evaluation
Source: Nanomaterials (Basel). 2022 Nov 28;12(23):4231. doi: 10.3390/nano12234231 (PMC9739509; doi:10.3390/nano12234231)
Supplement: Supplementary file 1 [file nanomaterials-12-04231-s001.zip › nanomaterials-2015222-Supplementary.pdf]

Electronic Supplementary Information

Morphology-Controlled *Green* Synthesis of Magnetic Nanoparticles Using Extracts of 'Hairy' Roots: Environmental Application and Toxicity Evaluation

Natalia Kobylinska [1,*], Dmytro Klymchuk [2], Olena Khaynakova [3], Volodymyr Duplij [4] and Nadiia Matvieieva [4]

[1] Dumansky Institute of Colloid and Water Chemistry, National Academy of Science of Ukraine, 42 akad. Vernadskoho Blvd., 03142 Kyiv, Ukraine
[2] Kholodny Institute of Botany, National Academy of Science of Ukraine, 2 Tereshchenkivska Str, 02000 Kyiv, Ukraine
[3] Faculty of Chemistry, University of Oviedo, 8 Julián Claveria Av., 33006 Oviedo, Spain
[4] Institute of Cell Biology and Genetic Engineering, National Academy of Science of Ukraine, 148 Zabolotnogo Str., 03143 Kyiv, Ukraine
* Correspondence: kobilinskaya@univ.kiev.ua, Tel.: +38-050-411-6375

.

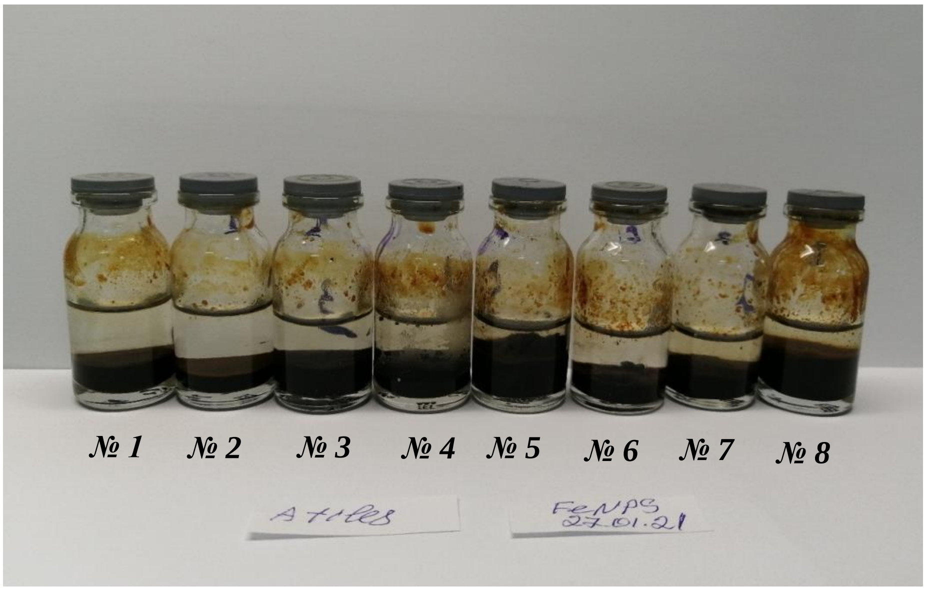

Figure S1. Biosinthesized MNPs using EtOH extract of *Artemisia tilessia* (control leaves (1) and roots (2), and "hairy" roots (3-6)) and $FeCl_3/FeSO_4/CoCl_2$ mixture at pH 9.

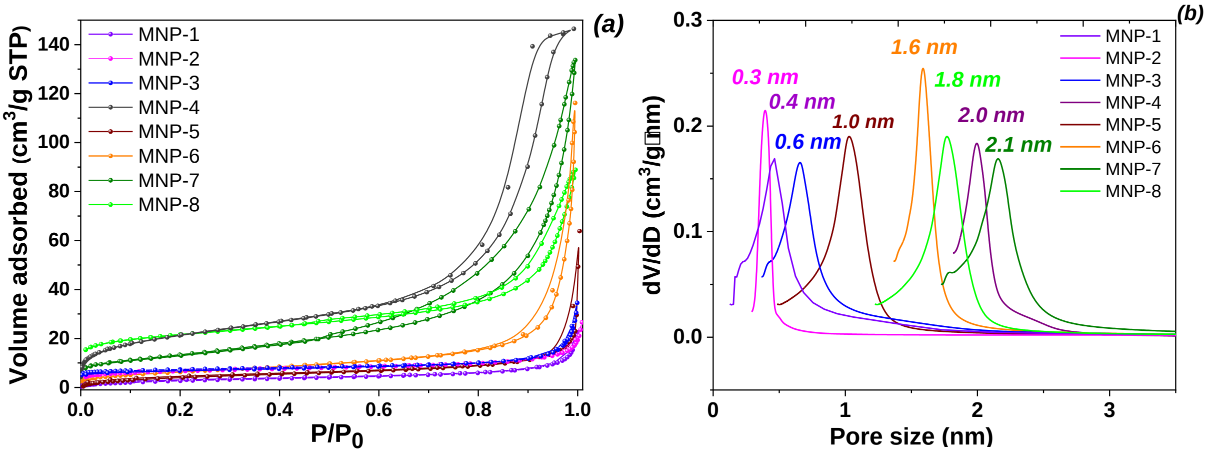

Figure S2. N_2 ad/desorption isotherms (**a**) and pore size distributions (**b**) for obtained magnetic samples.

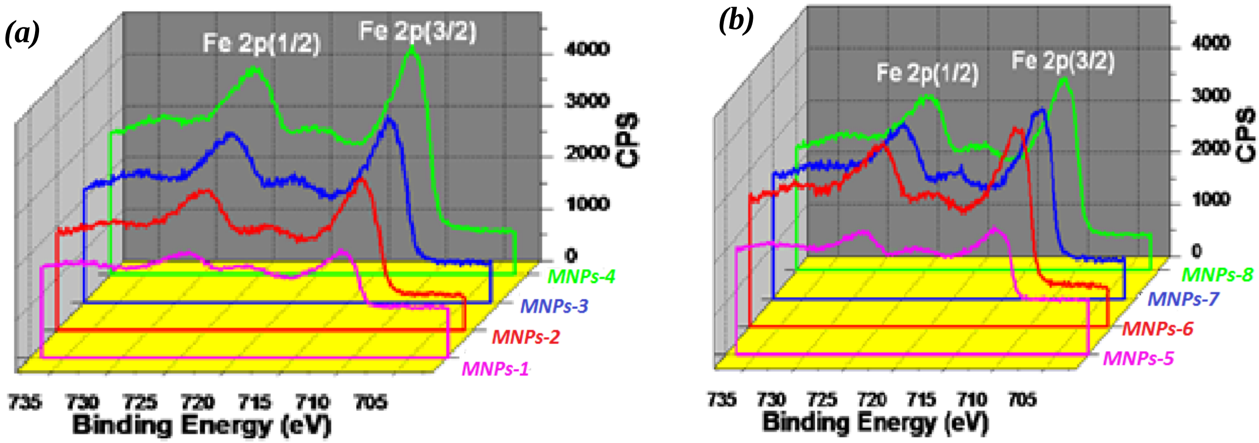

Figure S3. High resolution XPS Fe2p spectrum of as-biosynthesized magnetic samples: (**a**) – MNP-1, MNP-2, MNP-3 and MNP-4; (**b**) – MNP-5, MNP-6, MNP-7 and MNP-8.

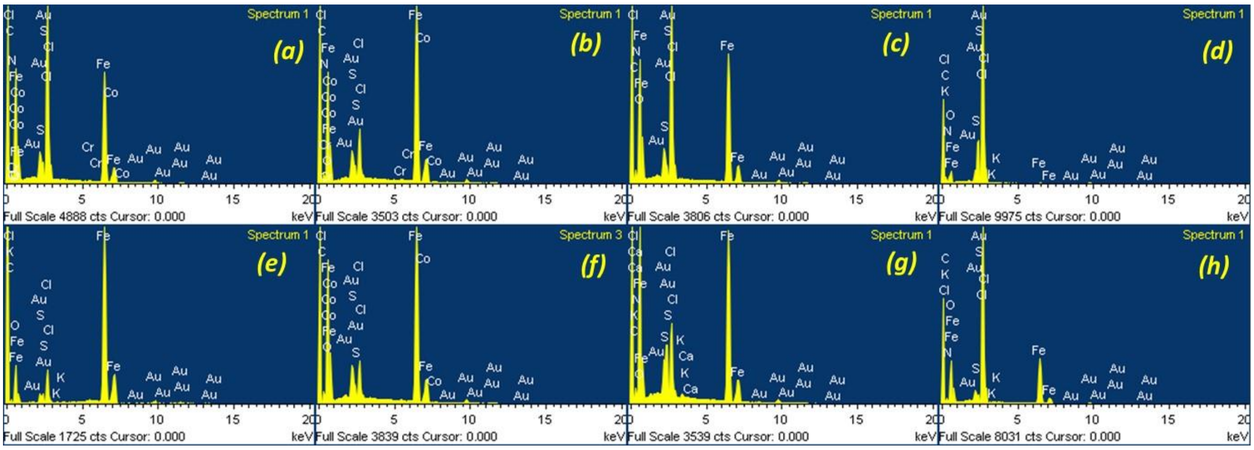

Figure S4. EDX spectra combine with microanalysis of as-prepared MNPs: (**a**) – MNP-1; (**b**) – MNP-2; (**c**) – MNP-3; (**d**) – MNP-4, (**e**) – MNP-5, (**f**) – MNP-6, (**g**) – MNP-7 and (**h**) – MNP-8 samples.

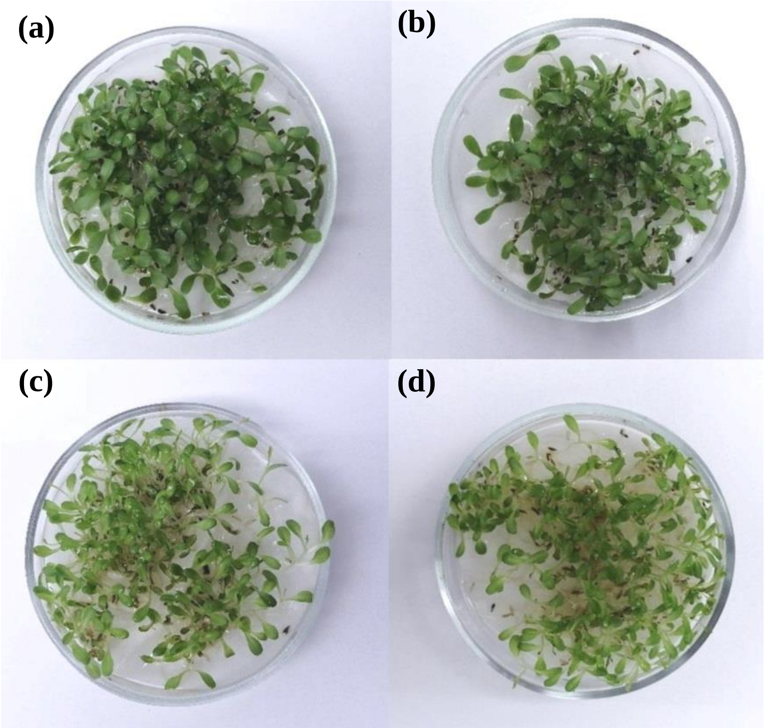

Figure S5. *Cichorium intybus L.* and *Lactuca sativa L.* control plants (**a**,**c**) and pretreated by MNPs (**b**,**d**).
